# Barriers to Point-of-Care Testing in India: Results from Qualitative Research across Different Settings, Users and Major Diseases

**DOI:** 10.1371/journal.pone.0135112

**Published:** 2015-08-14

**Authors:** Nora Engel, Gayatri Ganesh, Mamata Patil, Vijayashree Yellappa, Nitika Pant Pai, Caroline Vadnais, Madhukar Pai

**Affiliations:** 1 Department of Health, Ethics & Society, Research School for Public Health and Primary Care, Maastricht University, Maastricht, The Netherlands; 2 Institute of Public Health, Bangalore, India; 3 Division of Clinical Epidemiology, Department of Medicine, McGill University and McGill University Health Centre, Montreal, Canada; 4 McGill International TB Centre, Department of Epidemiology & Biostatistics, McGill University, Montreal, Canada; Vanderbilt University, UNITED STATES

## Abstract

**Background:**

Successful point-of-care testing, namely ensuring the completion of the test and treat cycle in the same encounter, has immense potential to reduce diagnostic and treatment delays, and impact patient outcomes. However, having rapid tests is not enough, as many barriers may prevent their successful implementation in point-of-care testing programs. Qualitative research on diagnostic practices may help identify such barriers across different points of care in health systems.

**Methods:**

In this exploratory qualitative study, we conducted 78 semi-structured interviews and 13 focus group discussions in an urban and rural area of Karnataka, India, with healthcare providers (doctors, nurses, specialists, traditional healers, and informal providers), patients, community health workers, test manufacturers, laboratory technicians, program managers and policy-makers. Participants were purposively sampled to represent settings of hospitals, peripheral labs, clinics, communities and homes, in both the public and private sectors.

**Results:**

In the Indian context, the onus is on the patient to ensure successful point-of-care testing across homes, clinics, labs and hospitals, amidst uncoordinated providers with divergent and often competing practices, in settings lacking material, money and human resources. We identified three overarching themes affecting point-of-care testing: the main theme is ‘relationships’ among providers and between providers and patients, influenced by the cross-cutting theme of ‘infrastructure’. Challenges with both result in ‘modified practices’ often favouring empirical (symptomatic) treatment over treatment guided by testing.

**Conclusions:**

Even if tests can be conducted on the spot and infrastructure challenges have been resolved, relationships among providers and between patients and providers are crucial for successful point-of-care testing. Furthermore, these barriers do not act in isolation, but are interlinked and need to be examined as such. Also, a test alone has only limited power to overcome those difficulties. Test developers, policy-makers, healthcare providers and funders need to use these insights in overcoming barriers to point-of-care testing programs.

## Introduction

Global health experts see immense potential in point-of-care (POC) tests to reduce delays in diagnosing and initiating treatment for diseases like tuberculosis (TB) [1–4], HIV [5, 6], syphilis [7] and malaria [8]. However, the mere availability of rapid or simple tests does not automatically ensure their adoption or successful scale-up as a POC test. Sometimes rapid tests are not implemented rapidly and instead patients might be sent home and told to come back for test results. Rapid tests get misused or underused [9], they require additional infrastructural, financial, and operational support [10] or their results are not used to impact treatment decisions in a timely manner [11].

We have previously argued [12] that a range of barriers may impede successful implementation of POC testing: economic (e.g. high costs and kick-backs), regulatory (e.g. poor quality products), and policy-related (e.g. uncertain policy recommendations for use of POC tests), as well as user/provider perceptions and cultural barriers. We have also suggested that technology alone does not define POC testing; how tests are put to use in POC testing programs matters [12]. What makes diagnostic technologies POC tests is their successful use at the point of care in ensuring a timely and rapid completion of the test and treat cycle in the same clinical encounter or at least the same day [12, 13].

In order to be able to successfully develop, validate, and scale-up diagnostics that ensure such POC continuums, more research, especially qualitative research, is needed that examines diagnostic practices on the ground and their implications for the completion of test and treat cycles across different points of placement within healthcare systems. Yet, to our knowledge, such studies are currently limited.

Qualitative and quantitative survey-based studies have examined clinicians’ attitudes towards POC testing as a potential indicator for its uptake. The most common concerns raised by clinicians were test accuracy, over-reliance on tests, undermining of clinical skills, cost, limited usefulness [14], need for training and required counselling [15], as well as lack of time, interruption of workflow and complexity [16]. Yet, clinicians’ attitudes might not reflect their daily diagnostic practices or capture systemic challenges for POC testing across the healthcare system.

Studies on the social processes of diagnosis [17, 18] mainly focus on treatment seeking behavior of patients. They show how stigma and disease perceptions influence healthcare seeking and diagnosis [19–21], reasons for delay in healthcare seeking [20, 22–25], and what it means to live with a particular diagnosis [26–28]. Such studies generate important results and more studies into pathways to diagnosis are needed. Yet, very few studies provide insights into social and health system contexts in which providers, health facilities and their diagnostic processes operate and how these influence testing practices [8, 29–32]. For example, HIV testing and referral practices for patients with TB in India are influenced by established national guidelines, partnerships with other providers, concerns over retaining patients and contrasting philosophies of public and private healthcare [33].

The material dimensions of diagnosis such as the test platform, reagents and supplies, the actors involved, their relations and the socio-cultural context in which testing, screening, confirmation and/or diagnosis is happening are invariably interlinked. Based on such an understanding, several authors have highlighted the additional efforts, such as infrastructure, training, maintenance, regulation and monitoring, that are required to successfully implement a diagnostic test, e.g. genetic testing [34], ultrasound [35], HIV [36], malaria [9], heart disease [37] and multi-drug resistant TB [38].

Almost all of these existing studies are single-disease focused. Yet, diagnostic tests are not conducted in isolation. Testing needs to fit into a variety of existing daily work flow and care processes. Patients present at different levels of care, in clinics, health posts, labs or hospitals. They may have multiple or unspecific symptoms or syndromes, and may need several diagnostic tests. Some testing, such as monitoring glucose levels for diabetes, might be conducted at home by patients themselves, while community health workers might test for some conditions during home or community visits. In clinics and hospitals, patients might be tested during consultations by doctors or nurses or be referred to laboratories on-site or outside their buildings in locations further away. In order to successfully develop, validate, and scale-up diagnostics that work in such complex and dynamic settings, more research on diagnostic practices on the ground across different healthcare settings and diseases is needed. We realize that different tests, diseases, or contexts may have unique ‘barriers’ to POC testing. For instance, stigma and confidentiality may be more important for HIV than malaria testing. Yet, our goal was to highlight the overarching barriers, such as policy, structural or process related barriers, that are important, regardless of disease, test or setting. We have identified no prior studies that have looked at the overarching challenges in POC testing programs and how testing different diseases at the same time produces additional challenges.

To address this knowledge gap, we conducted exploratory qualitative research on current diagnostic practices and challenges in five settings in India (i.e. home, community, lab, clinic, and hospital), with a focus on infectious diseases of global importance (i.e. HIV, TB, malaria, syphilis, and hepatitis), in urban/rural and public/private set-ups, and across a variety of providers and patients. Our overall objective was to understand whether POC testing is happening and where, and if not, why it is not happening, and to identify major barriers to POC testing.

In a prior analysis [39], we showed that successful POC testing hardly occurs in any of the five settings in India. In the current analysis, we examined in more detail *why* POC testing is not happening and what the major challenges are to diagnostic processes across these settings. For key stakeholders, such as test developers, policy-makers, public health officers and funders, it is important to understand these different processes and dynamics of POC testing for development of future tests and adaptation of existing ones [40].

## Methodology

### Setting and participants

The data presented in this paper were collected as part of a qualitative research project on barriers to POC testing. Data collection took place between January and June 2013 in Kadugondanahalli, one of Bangalore’s 198 administrative units, and Tumkur, a rural district in Karnataka (India).

India’s health system is characterized by medical pluralism, low government spending, high out of pocket spending and a large, unregulated private sector [41, 42]. Private providers range from highly qualified specialists to unqualified practitioners and local healers [43], and associated laboratory services are offered by large state of the art laboratory chains, medium sized facilities, and small neighborhood labs. They are largely profit-driven, diverse and lacking formal/official quality assurance or accreditation.

In line with our perspective on testing as an undertaking that needs to fit into a variety of care processes, happens across various healthcare settings, and often involves unspecific syndromes or symptoms, we included a range of different healthcare providers across settings. This was deemed important to capture the highly pluralized Indian health system, different diagnostic practices and referral arrangements. The urban study setting is a predominantly poor neighborhood, including one area that is considered a slum, with a population of more than 44,500 individuals spread over 0.7 square kilometers consisting of migrants from other Indian states as well as locals. Available healthcare services in the area include two government health centres that provide outpatient care and outreach services, and 32 private providers from various systems of medicine including allopathy, Ayurveda, yoga, Unani, Siddha and homeopathy. The rural setting is located 70 km outside Bangalore with an estimated population of 2.7 million. The area includes a dominant private sector with providers ranging from informal to highly specialized ones, as well as a public district hospital, nine sub-district hospitals and 140 primary health centres (PHCs).

A total of 78 semi-structured interviews were conducted to investigate diagnostic practices with healthcare providers (doctors, nurses, specialists, traditional healers and informal providers) across different settings, patients, community health workers (CHWs), test manufacturers, laboratory technicians, program managers and policy-makers. Participants were approached face-to-face or by telephone and purposively sampled to represent the five settings of hospitals, peripheral labs, clinics, communities and homes, in both the public/private sector and rural/urban setting (see Table 1). Additionally, 13 focus group discussions (FGDs) were conducted to address challenges of diagnosing in a particular setting in-depth, share experiences and solicit opinions on what an ideal POC test should look like with select actors of each setting. These included TB and diabetic patients, CHWs, laboratory technicians, TB program staff and medical officers at public clinics and public hospital nurses. Participants were selected on a convenience basis to represent the different settings. We aimed to have two FGDs per setting. However, especially in the laboratory and clinic setting, FGDs were difficult to convene as lab technicians and clinicians needed to be gathered from different locations. Additional FGDs were organized where possible. The total number of FGD participants was 94, with a median group size of 6.

**Table 1:** Participant overview per setting.

Setting	Type of participant	No. of interviewed participants (interview code)				Total interviews	No. of FGDs (FGD code)
		Urban Public	Urban Private	Rural Public	Rural Private		
**HOME**	Tuberculosis (TB); Diabetes Mellitus (DM); Typhoid (TP) patients	2 (TB patient #2, 4)	2 (TB patient #1, DM patient #1)	1 (TB patient #3)	2 (TB & DM patient #5, TP patient #1)	7	3 (FGD #4, 10—DM patients, FGD #5—TB patients)
**COMMUNITY**	Community Health Worker (CHW); Auxiliary-Nurse Midwife (ANM); Accredited Social Health Activist (ASHA); Link Worker (LW); Community Health Assistant (CHA)	0	0	2 (CHW #1, 2)	0	2	5 (FGD #2 –ANM, FGD #3 –ASHA, FGD #7 –LW, FGD #8 –ANM, FGD #13—CHA)
**CLINIC**	Specialist doctor (SP); Hospital Manager (HM); Private practitioner (PP); Medical Officer (MO) Ayush Practitoner (AP)	2 (SP #6, HM #2)	6 (PP #2, 3, 4, 11, 13, AP #1)	2 (MO #1, 2)	9 (PP #1, 6, 7, 8, 9, 10, 12, 14, 15)	19	1 (FGD #6—MO)
**PERIPHERAL LAB (stand-alone)**	Lab technician (LT); Lab Material Distributer (LMD);		4 (LT #6, 7, 8, LMD #1)		10 (LT #3, 4, 10, 11, 12, 13, 14, 15, 17, 18,)	14	1 (FGD #9-LT)
**PERIPHERAL LAB (attached to clinic)**	Lab technician (LT); Lab Manager (LM); Lab Specialist (LSP)	1 (LT #5)	1 (LM #2)	3 (LT #2, 19, 20)	3 (LT #1, LSP #4, 5)	8	
**HOSPITAL**	Specialist provider (SP)	1 (SP #14)	5 (SP #8, 10, 11, 12, 13)	4 (SP #1, 2, 3, 15)	3 (SP #7, 9, 16)	13	0
	Hospital Manager (HM); Program Officer (PO); Private practitioner (PP)	0	1 (PP #5)	5 (PO #1, 2, 3, 4; HM #1)	0	6	2 (FGD #11, 12—TB PO)
	Staff Nurse (SN)	0	0	5 (SN #1, 2, 3, 4, 5)	0	5	1 (FGD #1-SN)
	Lab technician (LT); Lab Manager (LM)	0	3 (LT 9#, LM #1, 3)	1 (LT #16)	0	4	0
**TOTAL**						78	13

**Home**: TB—tuberculosis; DM—diabetes mellitus; TP—typhoid patients. **Community**: ANM—auxiliary-nurse midwife; ASHA-accredited social health activist; CHA—community health assistant; CHW—community health worker; LW—link worker. **Clinic**: AP—ayush practitioner; HM—hospital manager; MO—medical officer; PP—private practitioner; SP—specialist doctor. **Peripheral lab**: LM—lab manager; LMD—lab material distributer; LT—lab technician; LSP—lab specialist. **Hospital**: HM—hospital manager; LT—lab technician; LM—lab manager; PP–private practitioner; PO—program officer; SN–staff nurse; SP–specialist provider.

### Data collection

The semi-structured interviews and FGDs were conducted jointly by Patil (MP) (a public health scientist and physician) and Engel (NE) (a social scientist). The topics explored included diagnostic processes and their challenges for the major diseases, understanding of diagnosis, and visions for an ideal POC test. The interviews were adapted to the different participants based on initial questions about the kind of diseases study participants came across and the kind of tests they used. The remainder of the interview focused on the diseases and tests that participants were familiar with. The interviews examined diagnostic steps for each major disease, from ordering a test to acting on a result, including available material and capacities, turn-around-times (TATs), and referral processes of samples, reports or patients to other centres. Such a rich understanding of the daily diagnostic practices also helped to put participants’ views on POC testing into perspective and their visions for an ideal test (questions that were asked at the very end of each interview). The FGDs focused exclusively on challenges experienced when diagnosing. The moderator introduced the topic, explained the procedures and rules of the FGD and handed out sticky notes and pens. The FGD participants were asked to write down their major challenges in diagnosing diseases in their setting. These notes were collected, grouped and then the challenges were discussed one by one in the group. As most participating patients were illiterate, the patient FGDs were conducted without the sticky notes and instead patients were asked for their challenges one after the other. The moderator ensured that explanations and reasons for these challenges were explored as well as possible solutions. In a last step, participants were encouraged to discuss what criteria new POC diagnostic tests would need to meet in order to work in their setting. Interview and FGD guides were piloted and revised during the fieldwork to improve clarity of questions and translated into a local language (Kannada). Interviews and discussions were held in either English or Kannada, depending on the preference of the participants. Aside from 7 interviewees who refused, all interviews and discussions were digitally recorded, in addition to the note taker writing down main points raised, non-verbal communication and setting descriptors.

### Data entry and analysis

Audio recordings and notes were transcribed and, if applicable, translated into English. MP then checked and edited those files, which were then cross-checked by NE. Data analysis was done using Nvivo 9 (QSR International). The study team jointly devised a coding scheme, which was tested on a handful of varied interviews and further refined [44], and then coded the material in close communication with each other, further grouping material into emerging topics and themes in an iterative manner using thematic analysis [45, 46]. The analysis is based on writing memos and thick descriptions of diagnostic practices and challenges per setting and disease and of different subthemes, such as the steps involved in reaching a diagnosis (diagnosis seeking, ordering tests, conducting a test, handling results, testing/treating/attending, referral processes, testing/quality); interaction between providers; interaction with patients; human resources, money and material; patient pathways to diagnosis; ideal criteria of POC tests and modified practices of diagnosis and treatment. Furthermore, patterns and linkages between emerging themes and codes across settings, actors and diseases were examined. This happened through several cross-case searching tactics such as analyzing different understandings of concepts, iconic events or rituals and combing data by source [44, 45]. For this paper, we elaborated on the themes interaction among providers and interaction with patients (which was collated into the overall theme of relationships), infrastructure (human resources, money and material) and modified practices, as it became clear throughout the analysis that these were recurring barriers present across all settings, diseases, tests and steps involved. We chose examples from across the different settings and diseases to illustrate these. In this paper, professional roles are used to mask study participants’ identity.

### Ethics

The study was approved by the institutional ethics committee of the Institute of Public Health (IPH), Bangalore, India, and the ethics review board of the McGill University Health Centre (MUHC, 12-151-SDR), Montreal, Canada. Approvals for interviews and discussions were sought from district and local authorities as necessary. Study participants were provided with an information sheet explaining the objectives of the study and all but the participants of FGD #10 diabetic patients (who agreed to participation verbally) signed informed consent forms prior to participation.

## Results

Contrary to centralized diagnostic systems, such as South Africa, where most testing takes place in centralized public or private labs [47], the Indian diagnostic system is highly fragmented and largely unregulated. Laboratory-based testing takes place across a multitude of providers ranging from small, ill-equipped one room labs in public clinics to large hospital labs, from small private neighborhood labs with limited testing equipment, to medium sized facilities and state of the art laboratory chains. Private practitioners generally use services of small private labs nearby and rarely refer patients to the public sector hospitals. Sometimes patients in the public sector are referred to private labs. What is more, when patients are asked to obtain a diagnostic test they need to go to the lab themselves, provide a sample there, pick up results once available and return them to the doctor. This is true for all settings, small private clinics as well as large public hospitals. Patients are the carriers of samples, reports and communication between the providers. They need to navigate amidst a multitude of providers and often iterate between public and private providers and different levels of care. The system thus relies heavily on patients’ initiative to ensure successful POC testing. As we will show below, these journeys often start in the private sector (for rich and poor patients alike) and can be long, frustrating, exhausting or confusing. Many times patients give up or abandon the process.

Only a limited number of testing occurs outside laboratories, and this has been seen in quantitative surveys as well [48]. Among these, rapid tests have a high potential for ensuring the POC continuum which is currently not realized. In homes, for instance, glucometers are too costly for patients, while patients do not feel sufficiently empowered to read urine pregnancy test results and instead bring them to clinic doctors. Only a very limited number of tests are being used in the field by CHWs and their use is constrained by the lack of continuous supply of test kits. Within public clinics and hospital compounds, rapid card tests are used in the labs with too long TATs and backlogs [39].

**Fig 1:**
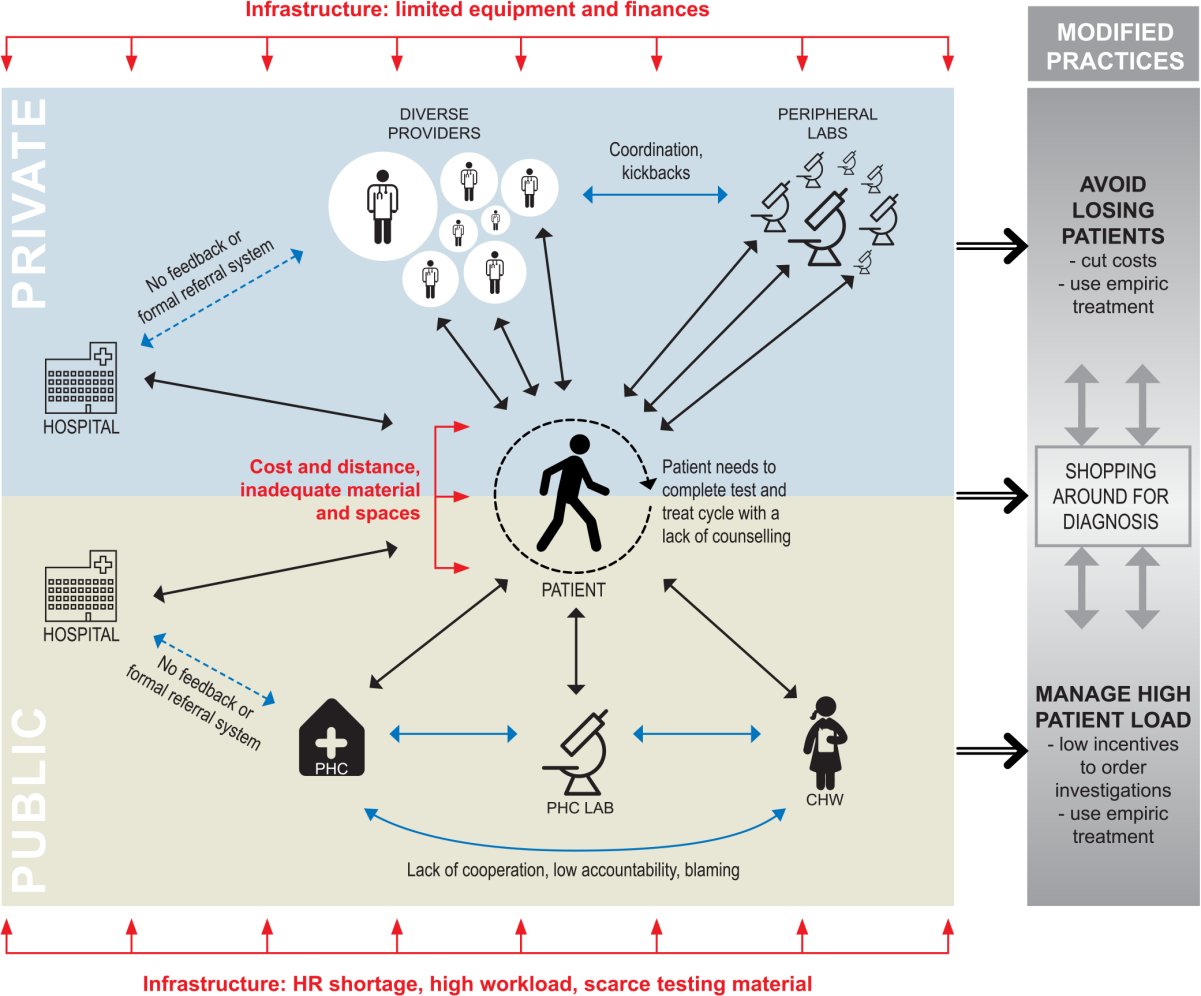
Barriers to POC testing: Relationships, infrastructure and modified practices. PHC- Public Health Centre. CHW–Community Health Worker.

Analyses of the interviews and FGDs suggested several barriers, often inter-related, to POC testing. Using thematic analysis in an iterative manner [45, 46], we grouped the results under three overarching themes that present challenges to the POC continuum (Fig 1): the main theme is *relationships (coordination*, *cooperation and patient initiative in seeking care)*. A cross-cutting theme is that of *infrastructure (material*, *money and human resources)* that impacts these relationships and in turn POC testing. The challenges emerging from relationships and infrastructure result in modified practices of providers and patients, highlighted in the last theme on *modified practices (empirical [symptomatic] treatment vs*. *investigation)*.

### 1. Relationships: coordination, cooperation and patient initiative in seeking care

The main theme emerging from the data is that interaction and coordination between providers, labs and patients matters for successful POC testing. Where there is interaction/coordination/cooperation between providers and patients, POC testing is more likely to take place. In all cases, the patient's initiative is relied upon to get tested and follow-up for results, irrespective of socio-economic background.

#### Coordination in the private sector

In the private sector, coordination between doctors and labs means that testing is often accomplished within the same day. A patient is seen in a clinic in the morning, goes to a nearby or in-house lab for tests and usually returns to the doctor with the results in the afternoon or evening. This occurs for the majority of tests such as for dengue, typhoid, malaria, platelet count, blood sugar and HIV. Labs and doctors have adjusted opening hours and try to cater to patients’ schedules (LT1-6, PP1, 6, 11). Private labs usually have the required human resources and facilities for what they offer, and aim to give results quickly to keep doctors and patients happy.

While these coordination efforts ensure a certain POC continuum, there are disruptions as well: kick-backs paid by labs to providers range between 20–40% for each test ordered and mean that doctors have an incentive to order wrong or unnecessary tests. For instance, the majority of TB patients we interviewed visited 4–5 private providers who all ordered a battery of blood and urine tests from small private labs to rule out typhoid and malaria fever, yet not a sputum test to diagnose TB. In the process, these patients lost considerable amounts of money and time and eventually ended up in the public sector, either on their own or through a referral (TB patient 1–5). A patient's knowledge of the arrangements between labs and doctors can lead to distrust, prompting them to switch providers (CHW1, 2, PP2, 10, 11, 12), or to fail to trust lab results and seek second opinions (LT17, 18).

For this reason, lab technicians believe that doctors fear losing patients who represent their livelihood and aim to control investigation processes. All lab technicians we spoke to made it clear that they have no say in what tests are ordered and are rarely consulted by the doctors (LT6, 14, 15, 17).

We will do as instructed by the doctor. Doctors will caution us against doing anything on our own or they will stop sending patients here. They will say ‘how dare you do everything yourselves’. We will advise the patient to go to the doctor for whatever problem he may have. (LT 7)

This also means that when some lab technicians know patients are being wrongly diagnosed/tested, they will not interfere to avoid alienating the doctor (LT6, 18, 19). But others do propose additional tests to the patient if they observe a need for it. For example, in cases of malaria, a lab technician suggests to add a platelet count to the total and differential blood count that doctors usually order. He says this saves the patient time and money, as in most cases the doctor would send the patient back to the lab with a request for platelet count (LT6).

Specifically for smaller private labs in poorer neighborhoods, infrastructural challenges can cause disruption of the coordination among providers. Limited equipment and space causes backlogs in testing samples and requires routine batch processing (LT3, 6, 15, 16). As a result, morning rush hours for diabetes patients (testing fasting glucose) delay TATs in these settings and threaten the coordination of opening hours with private providers (LT17-18).

The modes of coordination in the private sector ensure a certain POC continuum. These arrangements are threatened by disruptions such as kick-backs causing distrust between providers and patients, power relationships between doctors and lab technicians, limited lab equipment and patients’ financial and transportation constraints.

#### Lack of cooperation in the public sector

In the public sector, a lack of coordination among clinic staff (FGD9 LT; LT2), largely influenced by human resources shortages, fosters a culture of shifting blame among nurses, doctors, lab technicians and CHWs for delivering poor quality work. The consequences are delays and disruptions in POC continuums and unwillingness to take on more testing.

Human resources shortages: Most public clinic doctors and staff we spoke to report a grave mismatch between the population covered by a public health centre (PHC) and the human resources available, especially in hard to reach places (MO1, 2, PO1-4, FGD6 MO, FGD9 LT, LT2). Some medical officers might see 90–100 patients per day. Understaffed PHCs lead to an unequal distribution of responsibilities, over-worked and insufficiently supported personnel and delays or disruptions in POC continuums. A lack of personnel to clean lab equipment and transport samples or test results, for instance, creates backlogs, delays testing, frustrates lab technicians and discourages doctors to order investigations (LT 5, 19, 20, FGD 9 LT, MO1, 2; PO1, 3).

Laboratory technicians are overburdened in busy PHCs and complain about lack of assistants and their replacement when on leave. They say that the extensive testing for malaria which amounts to up to 900 slides per month as per the guidelines of the malaria control program is unnecessary in most areas and delays running other tests (LT2, FGD9 LT). At the same time, the cost of time/human resources-saving technologies, for instance malaria rapid tests in PHCs (MO1, 2, PO2, PP2, LT8) or rapid card tests in public hospital labs (SP1), are often too high for the limited budget of public sector labs faced with competing priorities.

High workload and lack of support and accountability: All public health providers complained about high workload and missing support with implications for time or willingness to do POC testing. PHC staff are burdened by record keeping for national disease programs such as the malaria, TB, HIV/AIDS control or reproductive and child health program (FGD8 ANM). A program officer and a lab technician illustrate:

They [medical officers] are loaded with programs, financial work, administrative work, that training, this training, so they will not have time [for testing patients]… (PO3)

There are three registers to fill in for malaria. For each patient, three times to write the same thing is very boring and takes too much time. (LT19)

Similarly in the public hospitals, some of the nurses we spoke to were in charge of two wards each. The extra work makes them unwilling to take on more testing besides sugar testing with a glucometer, blood pressure and taking malaria smears, even though immediate results could boost confidence and job satisfaction (SN1, FGD1 SN).

CHWs, complained about a lack of support to accomplish their diagnostic work. They face irregular and lower rates of remuneration, lack transport to (remote) communities, or incur risks to personal safety when dealing with domestic violence and alcoholism among patient families (FGD3 ASHA). CHWs in India mainly do symptom screening and collect malaria blood smears and sputum samples for TB microscopy testing. Yet, they are often seen as important users of future POC tests [49].

A laboratory technician laments the lack of cooperation within the PHC necessary to make POC testing work in situations of scarcity:

… it is not as if we are one group, the ANMs are separate, staff nurses are separate, lab separate, everybody is separate. If we request somebody to help us when they are free they say “we are not lab technicians.” There are so many people working but nobody is ready to support us.” (Participant3, FGD 9 LT)

Culture of blame and mistrust: These frustrations, along with the unequal distribution of responsibilities and over-worked, insufficiently supported personnel, discourage cooperation among public sector staff and foster a culture of blame. The different actors blame each other for poor quality of sample collection or laboratory work, and for inadequate numbers of investigations ordered to reach targets of disease control programs (LT2, 5, CHW1, 2, FGD13 CHA, FGD7 LW). A lab technician in a public clinic voices his suspicion of the quality of blood smears and sputum samples for malaria and TB testing that CHWs deliver:

I do not have much confidence in field workers. They are working only to achieve their targets. (LT19)

A TB program officer blames the need to reach targets for the low quality TB samples she receives from clinics:

They send samples because they are target oriented. So at the end of each month, because next month we will be reviewing the progress of the previous month, doctors, staff, field workers they refer lots of cases, even if it’s not a good [valid] case. … Not everyone, some doctors are very dedicated [to] sending quality samples. (PO3)

Similarly, some doctors who are under pressure to meet targets for collecting malaria blood smears or TB sputum samples, refer patients without symptoms to labs (LT19, PO3).

CHWs in an urban clinic take malaria smears, conduct pregnancy tests, create awareness, bring patients with fever and persistent cough or signs of jaundice to the centers. They argue that their role in the diagnostic process is not sufficiently acknowledged by higher level officers. Instead, these officers suspect them for not doing their work and therefore monitor or scold them, but never enquire about their problems or the feedback they bring from the community (FGD7 LW). For CHWs who are not formally trained health staff and only paid a stipend, trust from colleagues at the clinics and an appreciation of their work is a key motivating factor, a breakdown of which disrupts relations and working practices:

Staff nurses will not trust us. Only they will handle the delivery [of babies] even though the patient wants us there. (CHW1)

This lack of interaction, cooperation, accountability and trust among providers is detrimental to POC testing and is compounded by the lack of human resources, money and material and extends beyond the public clinic to other settings. Patient referrals between providers are often ad-hoc. They depend on personal relationships when doctors phone the next provider announcing the referral (MO2) or send personal thank you notes for referring patients. Additionally, there is hardly ever any feedback given to the referring doctor on whether and how the patient was helped (MO2, SP2).

#### Compromised patient’s initiative in seeking care

While cooperation or lack thereof among providers is a key aspect in POC testing, the onus to seek care and to complete test and treat cycles is ultimately on the patient. However, patients’ initiative is often compromised by a lack of counselling and trust in the interaction between practitioners and patients. Challenges with material and spaces as well as high cost of testing further discourage patients’ initiative and disrupt these social relations.

Lack of counselling: In our research, doctors tend to blame middle-class patients for their lack of health consciousness and adherence to advice, and poor/illiterate patients are deemed as incapable of learning (PP5-7, 10). Some doctors admit that they only involve patients in diagnostic and treatment decision-making if the patient or relatives are educated. The overall lack of counselling and communication by providers means patients lose trust, do not understand the importance of testing, do not return, or switch providers, especially if patients downplay symptoms or fear social stigma (DM patient1, TB & DM patient5).

Lab technicians and CHWs emphasize that counselling by doctors should extend to explaining the meaning and reasons for tests, the importance of collecting quality samples, the need to wait for results, adhere to a course of treatment, follow up testing and to act on results. According to them, this is especially important for diseases requiring antibiotics, and for TB or unconfirmed causes of fever, such as suspected dengue. It is also important to ensure that patients understand that tests alone will not cure, but that treatment is necessary (LT15, 17, 20, FGD13 CHA).

However, in public clinics and hospitals, the practice of counselling is limited to a protocol for stigmatized diseases like HIV. Generally, if test results are negative, patients are not contacted in the public sector, leaving some to wonder whether tests were conducted at all and diminish their trust in the health system (CHW1, 2, FGD13 CHA). In the private sector, counselling is not necessarily done for HIV either. Private doctors send patients with positive results directly to the government HIV/AIDS program. In one case, a private doctor used the rapidity and ease of use of the HIV rapid test to circumvent counselling or approval for testing. He conducted the test without a patient’s knowledge to not scare the patient away (PP5).

Due to the lack and particular nature of provider-patient interaction, it is not surprising that patients often iterate back and forth between different providers and different levels and sectors of care, either on their own or through referrals (TB & DM patient5, TB patient4, FGD13 CHA, FGD8 ANM, FGD4 DM). These iterations can delay testing. The delays are compounded by patients who do not reveal their entire history and doctors who lack time to take detailed histories (SP2, 9, 10, 14, PP11, 15).

Lacking material and inadequate spaces: Along with a lack of counselling for infections other than HIV, we find that infrastructural challenges, such as a lack of material in public clinics and the particular spaces associated with testing, can also both compromise a patient's initiative in seeking care and cause patient attrition.

Lack of material was most evident in public clinics and labs. Testing for a range of diseases was challenged by absent or poorly equipped laboratory facilities lacking microscopes, ultrasound and X-ray machines, inadequate space in labs and wards with no consultation areas that offer privacy, insufficient infrastructure for transportation of samples and personnel and lack of tests, equipment, and consumables for tests such as HIV, dengue, pregnancy or typhoid (MO1, 2, PO1-4, LT19, 20). A lack of materials either delays TATs or means patients have to be sent for testing elsewhere. This creates a disruption in the POC continuum and in the delicate balance of trust established between health providers and the community (MO1, 2, P01-4, FGD9 LT).

For instance, limited availability of or out-of-stock pregnancy kits at public clinics prevented CHWs from performing tests at camps or in households where testing can be more convenient or confidential for patients (CHW1, 2, FGD2 ANM). Equipment shortages at the doorstep and in the clinic send a bleak message to patients and disrupt social relations and trust (LT19, FGD3 ASHA, FGD13 CHA). A medical officer explains:

Often we do not get those [rapid test] materials, [so] we have to send them [the patients] away, refer them to another hospital or they go to private. If funds are there, we will purchase [the test material] otherwise it will get exhausted in 6 months, after that it becomes difficult to do the tests. I have to put money from my own pocket to purchase and the next year I take it from NRHM [National Rural Health Mission, a government scheme]. (MO1)

Often patients have to be referred to higher diagnostic centres at district or state level hospitals which can be as far as 100 km away (MO1, 2, FGD13 CHA). While some patients do come back to the PHC for treatment after being referred for diagnosis elsewhere, for instance for follow-up testing for TB or HIV, there is no formal, transparent and regulated referral system.

The particular space and infrastructural set-up of facilities can cause patient attrition, even when distances are shorter. In hospitals without a centralized laboratory, patients getting tested for typhoid, HIV and TB, for example, need to provide samples at three different labs, wait for results at each and queue to see the doctor again in the late afternoon or early evening. Time required and opportunities for getting lost and abandoning the process are therefore increased (SP1, 2, HM1).

High cost of testing: The costs incurred by patients to get tested are hampering their initiative to seek care across all settings. It should be noted however that most of our data were collected in poorer areas.

The cost of most rapid card tests can be high in relation to their setting of use, particularly due to the cost of reagents. Glucometers used at home, for instance, cost USD24 and up with USD20 for 50 test strips (PP1, 3, 5, LT13, 14, FGD4 DM patients). Prices for rapid tests in small private labs range from USD2-12, compared to USD0.5–1.5 for older methods (LT1, 8, 15). For poor patients in both rural and urban areas, paying more than 2USD for just one test in the private sector is very costly., Especially, because they also need to pay for consultation fees, transport (from home, to clinic, to diagnostic centres), food and/or accommodation, drugs and often loss of daily wages (TP patient1, FGD4 DM patients). Furthermore, not all aspects of public sector healthcare are free. Patients often have to pay user fees and pay for drugs and selected investigations (LT1, 8, 15).

Costs and TATs for test results are interlinked: poorer patients cannot afford to wait too long or return to pick up results another day, but rapid tests are too costly for them. Cost of testing is increased if referrals are required. For patients, referrals cost time and money and are often de-motivating and frustrating. Testing facilities are far away, opening hours of public providers are not aligned and there is no chain of communication other than through the patient. Patients need to chase doctors who work in both public and private clinics or hospitals at the same time and are blamed for showing up at the wrong place (SP4-6, 11, 16, HM1, PP13).

If patients’ initiative to seek care and complete test and treat cycles is compromised by disrupted social relations to providers, inadequate material or spaces and high cost, patients can get lost in many test cycles occurring at different points of care across public and private sectors. Ultimately, these patients end up not getting treated or being treated incompletely.

### 2. Modified practices: empirical treatment vs. investigation

The specific social relations and infrastructural challenges result in modified practices by providers (avoiding losing patients) and patients (shopping around for a diagnosis). These modified practices often favor empirical (symptomatic) treatments over treatment guided by testing or lead to inappropriate or unnecessary testing.

#### Avoiding losing patients

Since the system relies heavily on patients’ initiative to ensure POC continuums, providers aim to make the medical visit more attractive or convenient for patients who pressure for fast results or no tests to cut costs.

In particular, private providers try to provide instant relief of symptoms through prescribing stronger antibiotics, steroid or pain killer injections or intravenous fluids or offering a bed while conducting lab tests (PP11, 15). To preserve their client relationships, private clinics and small private labs in poorer neighborhoods further prefer conventional, slower diagnostic methods rather than costly rapid tests and try to cut time by prioritizing testing for patients from far away, batching and alignment of opening hours to other providers, bus schedules, etc. (SP9, 13, PP5).

In the public sector, in settings characterized by high workload and lacking infrastructure doctors have few incentives to order investigations. Consultation times are very short [50] and patient volumes very high. Doctors often have no time and no privacy for detailed history taking, for consulting and ordering investigations. Some simply have no nearby or in-house lab facilities available as discussed above. Also, perception of poor quality lab testing may force doctors to rely on their own clinical acumen (MO1,2, PO3).

The presence of functioning referral systems between providers further determines how many and which tests are ordered and how long it takes for results. If TATs are too long, doctors revert to empirical treatment for patients who cannot wait in order to avoid losing the patient. We found this for instance in cases of dengue, malaria and suspected TB. This is especially true in urban private clinics where patients can switch providers easily and in rural hospitals where patients come from far away villages (SP2, 16, PP1, 11). Furthermore, easy availability of antibiotics due to poor regulation results in widespread antibiotic abuse without diagnostic confirmation [50].

#### Shopping around for a diagnosis

According to providers, patients also modify their behavior depending on the setting. Patients may shop around for private providers to confirm a diagnosis and before agreeing to (more) investigations. This is particularly the case if patients’ initiative to seek care has been compromised by a lack of counseling, inadequate material or spaces and high cost, as discussed above. As a result, diagnostic delay is accumulated, especially if each new doctor is starting from scratch with empirical treatment (PP5, 7, MO1).

In the costly private and the crowded public health clinics, for instance, costs and waiting times dissuade middle-class patients from seeking diagnostic services, rather than the lack of priority given to matters of health as cited by some doctors (SP9, 13, PP5). According to lab technicians, patients struggle with overcoming challenges posed by cost and distances, and those with limited education and finances sometimes fail to collect test results or act on them (LT17, 18). Some patients also pressure lab technicians into giving them a diagnosis based on the lab results to avoid the waiting times at the doctor. This is common in cases of suspected malaria, typhoid and blood sugar tests (LT3, 6, 17, 18). In hospitals, patients are more willing to agree and pay for investigations because they are sicker (PP5), have symptoms that have not been relieved in other settings and hospitals seem more trustworthy.

Shopping around for a diagnosis can mean both, to avoid referrals to other providers or to actively seek them. In order to avoid the cost associated with referrals, some patients decline getting tested or switch providers, some may feel better in the interim and others may succumb to their illness (MO1, 2, SP2, 7, 9). Poor and middle-income patients switch from the private to the public sector to access free drugs (e.g. for TB). When forced to retest, divergent test results that patients present from private labs can cause confusion and quarrels causing some patients to opt out and seek help elsewhere (PP1, 12, LT9, FGD6 MO).

To conclude, the preference of empirical treatment over investigations is both a consequence of the social relations and infrastructural challenges outlined above and a barrier to POC testing in and of itself. This means that even if new POC tests are introduced, they might not be used, as patients avoid spending time and money on investigations or doctors place more importance on empirical treatment than investigations in managing patients. Indeed, some of the available rapid tests for malaria and TB face this challenge [9, 51]).

## Discussion

POC testing in India is challenged by a variety of barriers that interact in a complex system of relationships, infrastructure and modified and dynamic practices of providers and patients. The onus is often on the patient to ensure completion of test and treat cycles across homes, clinics, labs and hospitals, amidst a multitude of public and private providers with divergent and often competing practices in settings lacking material, money and human resources. Since the system relies heavily on patients’ initiative to make POC testing work, counselling by providers about the various aspects of testing is a way to mediate diagnostic cycles. Functioning relations between providers and patients are thus a necessary but insufficient condition to make POC testing work. The relations among providers are equally important to test and treat cycles. These socio-cultural relations between actors, material aspects and diagnostic practices are intrinsically linked.

### Social relations critical to the POC continuum

While infrastructural challenges are an acknowledged challenge for scale-up of POC testing programs [52–54], relationships among providers and between providers and patients are rarely thought of as a key factor that impacts clinical outcomes associated with testing. Anthropological work on malaria rapid tests showed how the over-diagnosis of malaria in Tanzania is caused by multiple social influences, such as perceived expectations from colleagues, training in a context where the importance of malaria is strongly promoted, pressure to conform with perceived patient needs and quality of diagnostic support [55]. Similarly, malaria rapid tests in Cameroon were not used as expected, because the WHO guidelines disregard the social roles of test, treatment and health workers. In the same vein, our material shows that social relations among healthcare providers and between patients and providers are crucial to the POC continuum. This is true even if tests can be conducted on the spot and infrastructure challenges have been resolved. Social relations can prolong disease detection, disrupt test and treat cycles and reduce treatment adherence through weak referral system and because the onus is on patients to seek care. Social relations can also reduce confidence in each other’s quality of diagnostic work and integrity [56] or confidence in care offered at public and private clinics, for instance due to the lack of coordination in the public sector and kick-backs in the private sector.

As a result, patients iterate between public and private providers and between different levels of care, and diagnostic delays and repeat cycles of testing are increased often with suboptimal care and follow-up plans. This is particularly well reported in the case of TB, where patients get diagnosed after nearly 2 months and having seen 3 different providers with often detrimental effects on their health [57]. Attempts have also been made to cut delays and increase coordination among providers in diagnosing malaria by placing rapid tests at drug shops in Uganda [58]. The unregulated nature of the Indian private healthcare and diagnostic sector contributes to the lack of coordination among providers, as is widely recognized in literature on the Indian health system [41, 43, 56]. Our results also show that local forms of coordination among private doctors and labs, motivated by attempts to maintain client relationships, ensure POC continuums to some extent.

Challenges in *infrastructure* have a bearing on the relationships among providers and between providers and patients. These gaps put limits to the material, money, human resources and physical infrastructure that new tests rely on. They are currently most acute in the public clinic setting but also occur in hospitals and in the private sector. Challenges in infrastructure can disrupt social relations by affecting trust and blaming and can modify working practices by leading to a preference for empirical treatment, unequal distribution of responsibilities and disrupted coordination. The dominance of *empirical treatment over investigation*, for instance, is a well-researched and well-known problem in India and elsewhere [9, 59–61]. Our results demonstrate how the preference for empirical treatment is related to and enhanced through the specific relationships between providers, labs and patients and compounded by gaps in infrastructure.

Successful POC testing programs thus assume and need efficient social relations between providers, labs and patients. After all, the conduct of testing is not the essence of POC testing; it is the translation of the result into a clinical plan that makes the difference.

### Support needed for patients to complete diagnostic cycles

The relationship between patients and health professionals has been researched extensively [62–64], yet less so for the implications for diagnostic processes and functioning of diagnostic tests. In India, the providers rely on a patient’s initiative to seek care to complete diagnostic processes. These implicit ideas of a patient’s responsibilities, namely that the patient accesses care and is willing to go to the nearby lab and come back to the provider with the results, are embedded in the relationships between providers and labs. These modes of ordering [65] of diagnostic processes might be different in other countries and might be differently inscribed in diagnostic guidelines.

In the settings we studied, if a patient’s initiative is not supported, patients do not seek care, pick up results or adhere to advice. Instead, they may drop out, mistrust the provider or test results, have wrong tests done or lose money and time while their health deteriorates. This is not so much a matter of their attitude and willingness for testing as some providers would like to believe, but more often a matter of a lack of counselling and trust that characterizes the interaction between providers and patients. Absent explanations and counselling alongside diagnostic testing leave patients in the dark about their condition or the implications of the testing process. An exception is HIV testing, where consenting and counselling is deeply ingrained in the testing process in the public sector, but may not necessarily be adhered to in the private sector [57, 66]. Research on HIV counselling practices shows that it is crucial for seeking further care and treatment adherence [31, 32], while counselling practices differ across professionals and institutions [30] and are prone to power imbalances between patients and providers [29]. This applies to all diseases, not just HIV, and extends to how results, whether positive or negative, are communicated across settings.

Inadequate material and spaces, high cost of testing and particular modes of (un-) coordination between providers can further discourage patients’ initiative. This is not only true if diagnostic processes require samples or patients to be sent to different sites or healthcare providers, as they often do in India. It is also true for tests that can be conducted on the spot, such as rapid tests. These tests have a high potential for ensuring the POC continuum which is currently not realized [48]. In the current Indian context, rapid tests still rely on the patient’s initiative to seek care for confirmatory testing and treatment initiation and thus cannot overcome the challenges of infrastructure and relationships.

### Diagnostic technology can harm or support relationships

Diagnostic technology, such as rapid diagnostic tests, can harm or dilute these relationships further by allowing providers to circumvent counselling, explanations or approval for testing due to a test’s rapidity and ease of use for example. On the other hand, tests can support providers in instilling trust into the healthcare system. A diagnostic test that can be conducted at the doorstep can support the CHW in convincing patients to come to the public clinic. Yet, if done inconsistently, the same test can damage these relationships. Similarly, disappointments at the clinic with lack of facilities, human resources and functioning coordination and counselling undermine some of the work done by tests in the communities, for instance convincing patients to seek care. Having a test that is rapid and easy to use is not POC testing [12]; it must be deployed with the relationships, infrastructure and modified practices in mind.

Contrary to studies on attitudes towards POC testing of selected actors such as clinicians, our insights into diagnostic practices and processes across different points of care are able to explain why tests are likely not to be used in the way envisioned by the developers. Furthermore, we show how they interact with other priorities (targets and maintenance of clientele), existing relationships (kick-backs between private doctors and local labs, disrupted social relations in public sector and lack of trust from patients) and capacities (skills, human resources, equipment and cost) in each setting. Our focus on multiple diseases showed how different diagnostic processes at times compete with each other when creating backlogs in labs or when ordering investigations or budgeting reagents. Our results also show that a test alone has only limited power to change and circumvent those difficulties and worse, might at times encourage them.

Furthermore, specific barriers, such as human resources shortages, lack of testing material and lack of coordination among public providers, do not act in isolation but are interlinked. POC testing can thus only be understood when those dynamics are examined as a whole. This also means that no single actor or aspect can be held accountable for ensuring the POC continuum. For the development of new tests and adaptation of existing ones, test developers, policy-makers, healthcare providers and funders need to take into account each actor’s rationale for functioning in a dynamic and often resource-constrained POC setting. Otherwise, the true potential of POC testing will remain unrealized.
